# Global genetic diversity of the *Plasmodium vivax* transmission-blocking vaccine candidate Pvs48/45

**DOI:** 10.1186/s12936-016-1263-0

**Published:** 2016-04-12

**Authors:** Andres F. Vallejo, Nora L. Martinez, Alejandra Tobon, Jackeline Alger, Marcus V. Lacerda, Andrey V. Kajava, Myriam Arévalo-Herrera, Sócrates Herrera

**Affiliations:** Malaria Vaccine and Drug Development Center, Cali, Colombia; Facultad de Ciencias Médicas, Hospital Escuela Universitario, Universidad Nacional Autónoma de Honduras, Tegucigalpa, Honduras; Fundação de Medicina Tropical Dr. Heitor Vieira Dourado, Manaus, Brazil; Centre de Recherches Biochimie Macromoléculaire (CRBM), Institut de Biologie Computationnelle (IBC), CNRS, University of Montpellier, Montpellier, France; Institute of Bioengineering, University ITMO, Saint Petersburg, Russia; Caucaseco Scientific Research Center, Cali, Colombia; School of Health, Universidad del Valle, Cali, Colombia

## Abstract

**Background:**

*Plasmodium vivax* 48/45 protein is expressed on the surface of gametocytes/gametes and plays a key role in gamete fusion during fertilization. This protein was recently expressed in *Escherichia coli* host as a recombinant product that was highly immunogenic in mice and monkeys and induced antibodies with high transmission-blocking activity, suggesting its potential as a *P. vivax* transmission-blocking vaccine candidate. To determine sequence polymorphism of natural parasite isolates and its potential influence on the protein structure, all *pvs48/45* sequences reported in databases from around the world as well as those from low-transmission settings of Latin America were compared.

**Methods:**

*Plasmodium vivax* parasite isolates from malaria-endemic regions of Colombia, Brazil and Honduras (n = 60) were used to sequence the Pvs48/45 gene, and compared to those previously reported to GenBank and PlasmoDB (n = 222). Pvs48/45 gene haplotypes were analysed to determine the functional significance of genetic variation in protein structure and vaccine potential.

**Results:**

Nine non-synonymous substitutions (E35K, Y196H, H211N, K250N, D335Y, E353Q, A376T, K390T, K418R) and three synonymous substitutions (I73, T149, C156) that define seven different haplotypes were found among the 282 isolates from nine countries when compared with the Sal I reference sequence. Nucleotide diversity (π) was 0.00173 for worldwide samples (range 0.00033–0.00216), resulting in relatively high diversity in Myanmar and Colombia, and low diversity in Mexico, Peru and South Korea. The two most frequent substitutions (E353Q: 41.9 %, K250N: 39.5 %) were predicted to be located in antigenic regions without affecting putative B cell epitopes or the tertiary protein structure.

**Conclusions:**

There is limited sequence polymorphism in *pvs48/45* with noted geographical clustering among Asian and American isolates. The low genetic diversity of the protein does not influence the predicted antigenicity or protein structure and, therefore, supports its further development as transmission-blocking vaccine candidate.

## Background

Malaria is an infectious parasitic disease caused by the genus *Plasmodium* which is transmitted by bites of infected *Anopheles* mosquitoes. *Plasmodium falciparum* and *Plasmodium vivax* are the two most common malaria parasites in humans, however differing in their clinical presentation and geographic distribution. *Plasmodium falciparum* causes the most severe symptoms and higher mortality, mainly among children under 5 years of age in Africa. *Plasmodium vivax* generally causes milder disease, is significantly less life-threatening [1] and is widely distributed in the Middle East, Asia, the Western Pacific, and Central and South America [2]. Despite global efforts to control malaria transmission resulting in a significant decrease in global incidence during the last decade, it continues to challenge public health systems, particularly in tropical countries. Current global malaria control strategies will greatly benefit from the development of an effective vaccine that interrupts malaria transmission among individuals of endemic communities [3, 4].

Proteins expressed by parasite sexual stages, namely gametocytes/gametes, could induce effective immune responses in the human host that would prevent gamete fertilization and zygote formation when ingested by the mosquito during a blood meal [5].

*PlasmodiumPs*48/45 proteins are expressed by male and female gametocytes/gametes during the parasite maturation process, and are therefore classified as pre-fertilization antigens [6]. These proteins belong to a family common to all *Plasmodium* species characterized by the presence of partially conserved domains containing six cysteine (Cys) amino acid residues that form one to three disulfide bridges, resulting in a specific tertiary structure [7, 8]. In *P. falciparum*, this protein (Pfs48/45) is expressed on the surface membrane of gametocytes [9] and is required for male fertility [6]. In addition, Pfs48/45 is necessary for production of high antibody titers in individuals living in endemic areas [10, 11] that can reduce ookinete production and induce transmission-blocking activity [12–14]. It is currently considered a potential target for development as a transmission-blocking vaccine. As other proteins expressed on sexual forms, Pfs48/45 is rich in Cys residues, which has made it difficult to express as a recombinant product with proper conformation [5, 15]. Pvs48/45, the homologous protein in *P. vivax,* was recently expressed in *Escherichia coli* and its immunogenicity was assessed in mice and *Aotus* monkeys. These studies indicated high immunogenicity in both animal models and the elicited antibodies displayed significant and reproducible transmission-blocking activity in ex vivo *P. vivax* membrane-feeding assays (MFA) [9].

Genetic diversity could generate antigenic polymorphisms, which in turn could induce changes in critical epitopes and hamper vaccine efficacy. Successful development of an effective transmission-blocking vaccine is likely dependent on an assessment of the degree of genetic diversity in *pvs48/45* among *P. vivax* parasite populations in malaria-endemic locations [16]. Although available data indicate a limited Pvs48/45 genetic polymorphism on a regional scale [17, 18], knowledge of the sequence polymorphism on a broader scale and its potential impact on vaccine development is needed. Here, a total of 282 *pvs48/45* sequences corresponding to parasites from eight countries from around the world were analysed for gene diversity to assess probable protein changes that could influence the immunogenicity and its vaccine potential.

## Methods

### Ethics statement

Blood samples used in this study were obtained from studies approved by the Institutional Review Board (IRB) of the Malaria Vaccine and Drug Development Center (MVDC) under the codes CIV-01-042009, CIV 08-102010 and CIV 009. Samples from volunteers were not linked to the identity of the donor. Written informed consent was obtained from each volunteer at enrolment. All volunteers were adults over 18 years of age.

### Origin of *Plasmodium vivax* samples

The genetic diversity of *pvs48/45* was studied among 60 *P. vivax* isolates from endemic regions of Brazil, Colombia and Honduras in Latin American (LA). The geographical origin of each of the LA isolates analysed in this study is listed in Table 1. Brazilian *P. vivax* isolates (n = 13) were obtained from patients attending a tertiary healthcare centre in Manaus (Amazonas State) [19]; Colombian parasite samples (n = 35) were collected in four different malaria-endemic regions described elsewhere [20]; Honduran samples (n = 12) were collected at the University Hospital located in Tegucigalpa, and its origin was determined to seven different localities throughout the country. Additionally, a total of 222 sequences reported to the GenBank and PlasmoDB originally from South Korea [17], North Korea [18], India and Indonesia [18], Thailand and Vanuatu [21], Mexico, Peru, Thailand and Myanmar (*Plasmodium vivax* Hybrid Selection initiative, Broad Institute [22, 23], and reference sequences from Brazil, India, Mauritania, and North Korea were used for comparison.

**Table 1:** Origin of Latin American isolates used for *pvs48/45* sequencing

Country	Department	Locality	N
Brazil	Amazonas	Manaus	14
Honduras	El Paraiso	Moroceli	1
	Olancho	Catacamas	2
		Juticalpa	1
	Francisco Morazan	Talanga	2
		Tegucigalpa (CMDC)	2
	Gracias a Dios	Pto. Lempira	1
		Wampusirpi	2
Colombia	Cordoba	Tierralta	10
	Nariño	Tumaco	10
	Valle del Cauca	Buenaventura	8
	Choco	Quibdó	7

### Extraction and purification of parasite genomic DNA

Parasite genomic DNA was extracted from filter-paper blood spots using 10 % Chelex 100 (Biorad, USA) and from EDTA-blood samples using the salting-out method [24], and were identified with two-letter codes according to the country of origin as follows: Colombia (Co), Brazil (Br) and Honduras (Hn), followed by two numerical digits. Subsequently, DNA was purified using the PureLink Genomic DNA Mini Kit (Life Technologies, USA), according to the manufacturer’s instructions. DNA samples were tested by real-time quantitative PCR (qPCR) to confirm infection with *P. vivax* and rule out *P. falciparum* co-infection as previously described [25].

### Amplification and sequencing of the Pvs48/45 gene in parasite samples from Latin America

Pvs48/45F (5′-GGAATAATTTCGACCACTC-3′) and Pvs 48/45R (5′- TCAGAAGTACAACAGGAG-3′) primers were designed to amplify the Pvs48/45 gene coding region (CDS annotation 1357 bp) from the *pvs48/45* of Salvador I strain (PlasmoDB Id: PVX_083235). Amplification was performed in 25 μL reactions containing 2–4 μL genomic DNA, 500 nM of primers Pvs48/45F and Pvs48/45R, and 0.5 units of HotStar HiFidelity Polymerase (QIAGEN, Valencia, CA, USA). Thermal cycling conditions were as follows: initial denaturation for 5 min at 94 °C, followed by 39 cycles of: 94 °C for 30 s, 55 °C for 1 min, 72 °C for 2 min, and a final extension step at 72 °C for 10 min. Amplification products were analysed by gel electrophoresis in 1.2 % agarose gels and purified using the High Pure PCR product purification kit, according to the manufacturer’s recommendations (Roche Diagnostics, Mannheim, Germany). Purified PCR products were sequenced using the Big Dye Terminator Kit v3.1 (Applied Biosystems, Austin, TX, USA) in an ABI-PRISM Avant 3100 Automatic Genetic Analyzer (Applied Biosystems, Foster City, CA, USA) using primers Pvs48/45F and Pvs48/45R. Each purified product was sequenced in both directions by a minimum of two independent sequencing reactions until quality base calling value scores >20 (predictive sequencing error rate per base of 1.00 %) were obtained [26], as assessed with the Sequencing Analysis Software v.5.3 (Applied Biosystems).

### Sequence assembling, alignment and statistical analysis

All sequences were assembled against the Sal I *P. vivaxpvs48/45* sequence reported in PlasmoDB (accession No. PVX_083235) using the reference assembler algorithm in Geneious 4.8.4 [27] and corrections made by manual inspection. The primers sequences were removed before the analysis. Consensus sequences were aligned using the Geneious alignment algorithm and the alignment was used to calculate the number of segregating sites (S), singleton sites, singleton variable (SV) sites, parsimony informative (PI) sites, haplotype diversity (Hd), nucleotide diversity (π), and the average number of pair-wise nucleotide differences within the population (*K*), number of synonymous (dS) and non-synonymous (dN) substitutions with DnaSP 5.10 package using the Jukes-Cantor model. DNA polymorphism was analysed with a sliding window of 100 bases and a step of 25 bases for a haploid genome [28]. To determine the influence of the geographical origin of the isolates on nucleotide diversity and DNA polymorphism, the same analysis discriminating between groups of samples from Brazil, Colombia and Honduras was performed. Output data were exported to Excel to plot overlapping π curves with standardized scales for the different groups. Numbers of synonymous (dS) and non-synonymous (dN) substitutions were compared by a two-tailed Z test (P < 0.05) using MEGA 6.06 [29] using the Nei and Gojobori’s method [30] with the Jukes-Cantor correction and its standard deviation determined by 1000 boot-strap replications [31]. Null hypothesis was dS = dN, thus indicating that the observed polymorphism was neutral.

### Phylogenetic and epitope conservation analysis

Tajima’s D test and Fu and Li’s D and F tests using *Plasmodium knowlesi pvs48/45* as an outgroup were performed on DNASP 5.10 to test for the neutral theory of evolution. Haplotypes reported in this study were compared to 132 *pvs48/45* sequences reported in GenBank for South Korea [17], North Korea [18], India and Indonesia [18], and reference sequences from Brazil, India, Mauritania, and North Korea and Sal I, same as described above, for a total of 252 isolates. In silico translated DNA sequences were aligned to the *Pfs*48/45 3D7 amino acid sequence (PFD3D7_1346700) to ensure the localization of the single nucleotide polymorphisms (SNPs) in the Cys-rich domains (CRD) I, II, and III were epitopes I, II, III and V as previously reported [12]. Phylogenetic analysis was performed with the 60 sequences reported in this study, and with the 49 sequences reported in GenBank.

### Immune epitope mapping

In order to determine the presence of linear B cell epitopes in the Pvs48/45 protein sequence and the possible influence of the SNP in the predicted epitopes, an in silico analysis of the entire sequence was performed. Epitopes were predicted based on hydrophilicity, accessibility, polarity, flexibility estimation, combined with secondary structure analysis (Kolaskar-Tongaonkar antigenicity prediction methods) [32].

### Molecular modelling of the 3D structure

Structural models of Pvs48/45 domains were built by using homology-based modelling. The known 3D structures of homologous proteins *Pf*12 from *P. falciparum* [33] and the immunodominant surface antigen from *Toxoplasma gondii* (Protein Data Bank IDs: 2YMO:A and 1KZQ:A) were used as templates. Sequence alignments were obtained by using a HHpred server [34], ClustalW program [35] and manual adjustment. Structural models were built using the HOMOLOGY module of Insight II program [36]. Resulting structures were subjected to energy minimization using the procedure implemented in the Discovery sub-routine of Insight II.

## Results

### Genetic polymorphism and amino acid changes

**Fig. 1:**
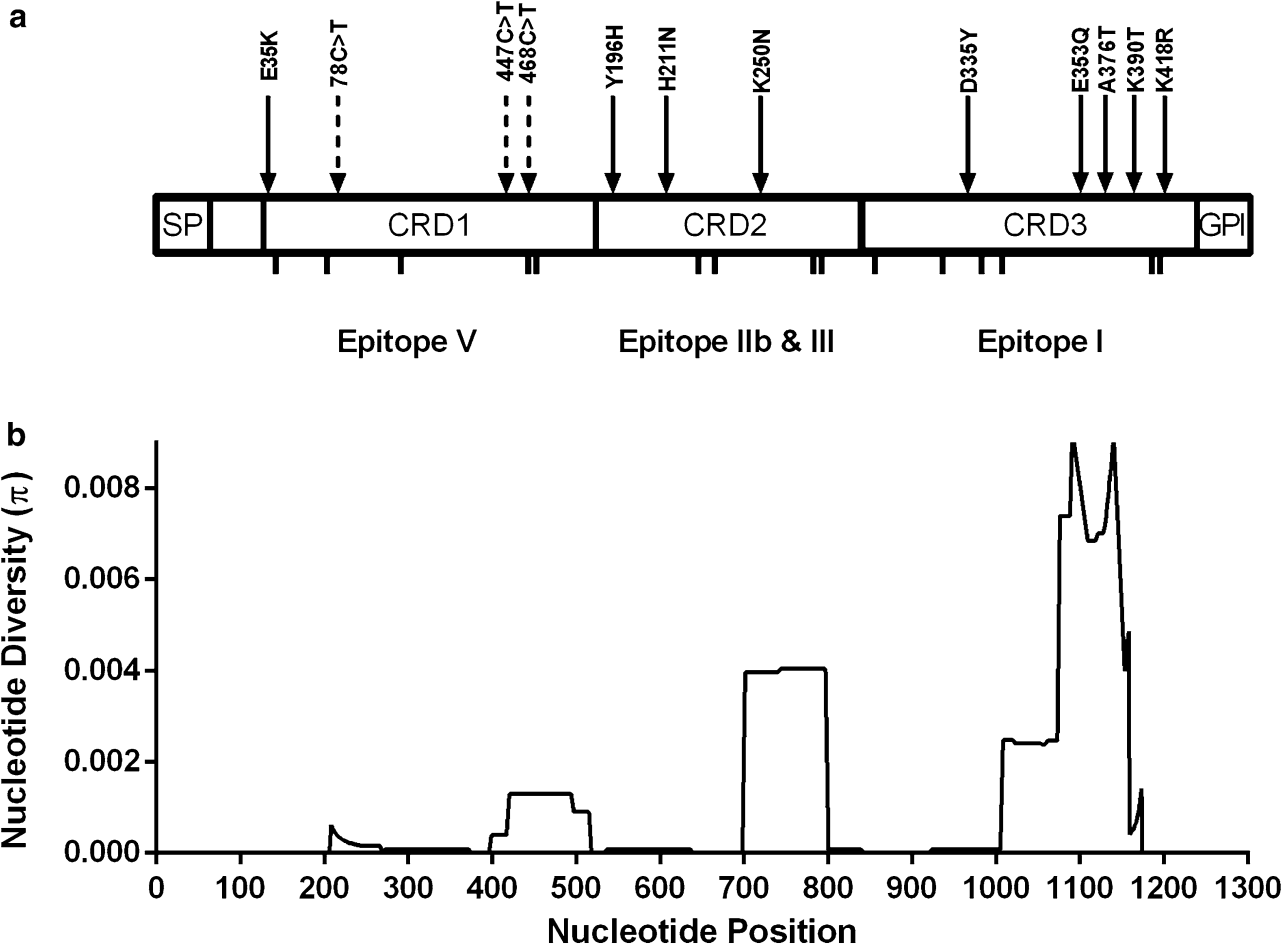
Nucleotide changes found and nucleotide diversity. **a** Structural architecture of Pvs48/45 reported by van Dijk [6]. Cysteine residues are depicted as small *black bars* under the three cysteine-rich domains (CRD), non-synonymous substitutions are shown by *solid arrows*, whereas synonymous substitutions by dashed arrows. **b** Sliding-window analysis of nucleotide diversity (π) along the Pvs48/45 gene using a length of 100 sites and a step-size of 25 sites

Results of the qPCR assay indicated that all samples were specific for *P. vivax* infections. PCR amplification of the Pvs48/45 gene-coding region showed a single band of ~1.3 kb in all samples, indicating an absence of size polymorphism. Comparison of the nucleotide sequences against the *pvs48/45* reference sequence (Sal I) showed that SNPs occurred at 15 positions. Nine of these mutations resulted in amino acid changes (non-synonymous substitutions) at positions E35K, Y196H, H211N, K250N, D335Y, E353Q, A376T, K390T, and K418R (Fig. 1a), while the remaining six had no amino acid variations (synonymous substitutions). Of the nine sites, two (H211N, K250N) were found worldwide, four in Asian isolates (E35K, D335Y, A376T, K418R), while the remaining three were country-specific (Y196H Thailand, K390T Peru, E353Q in Mexico, Peru and Colombia).

### Pvs48/45 gene haplotypes

Based on sequence analysis, the *pvs48/45* sequences were classified into seven haplotypes (haplotypes I–VII), in which three resulted from trimorphic polymorphisms, two from dimorphic polymorphisms, and one from a single polymorphism. None of these substitutions was common in all isolates. The amino acid substitution E353Q was found in haplotypes I–IV. The K250N substitution was found in haplotypes I, II, IV, V, and VII, while the H211N amino acid substitution was found in haplotypes I and V. Amino acid substitutions A108T, P264H, N324S, and K370N were only found in haplotypes IV, VI, VII, and V, respectively (Table 2).

**Table 2:** Amino acid changes found in the seven *pvs48/45* haplotypes in Latin American isolates

	Amino acid position							n	Frequency (%)
	108	211	250	264	324	353	370		
Sal I	T	H	K	P	N	E	K	68	25.1
I	–	N	N	–	–	Q	–	35	12.9
II	–	–	N	–	–	Q	–	9	3.3
III	–	–	–	–	–	Q	–	2	0.7
IV	A	–	N	–	–	Q	–	4	1.5
V	–	N	N	–	–	Q	N	83	30.6
VI	–	–	–	H	–	–	–	64	23.6
VII	–	–	N	–	S	–	–	6	2.2

### Global diversity of *pvs48/45*

**Fig. 2:**
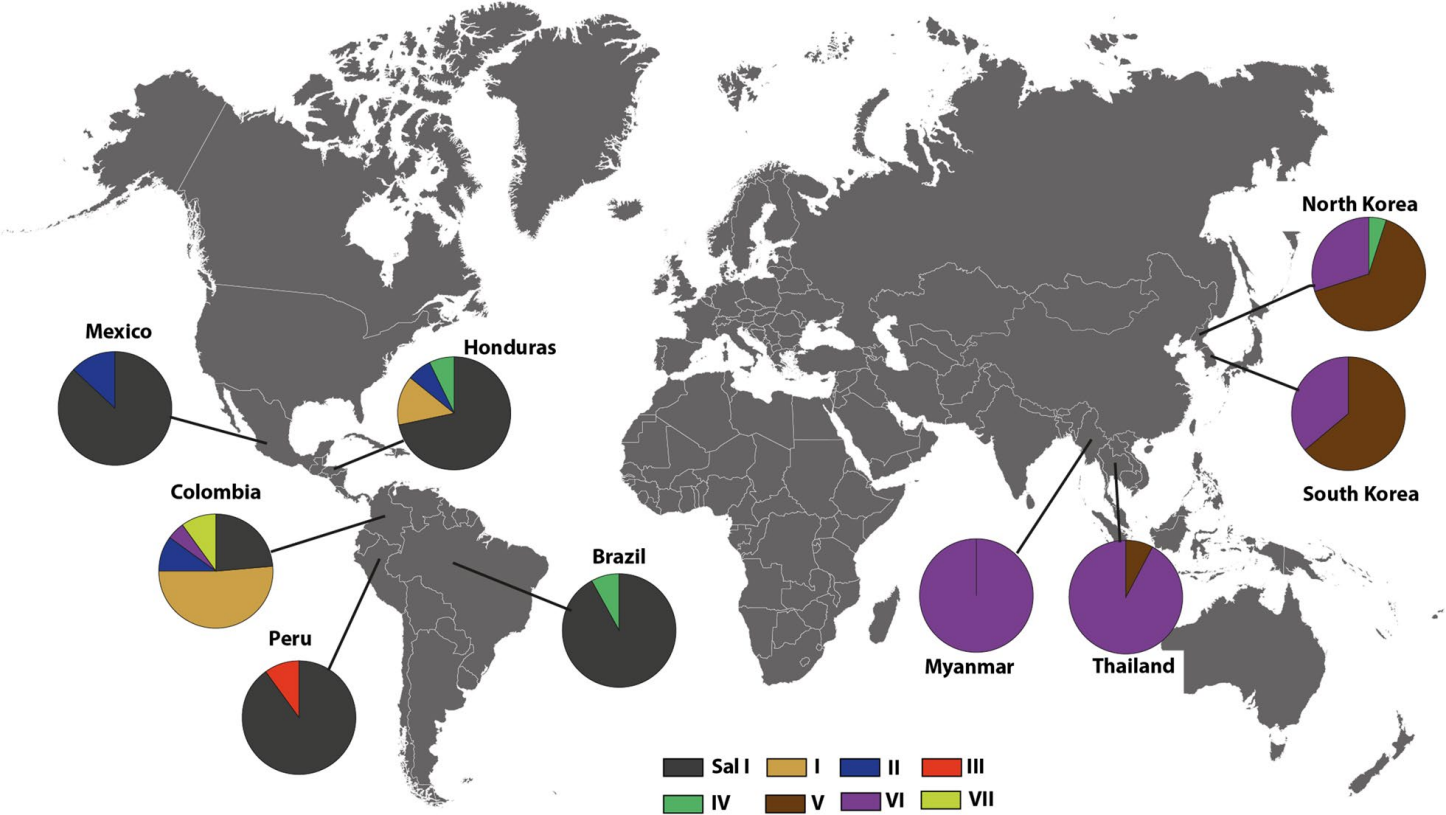
Distribution of *pvs48/45* haplotypes. Geographic distribution of *pvs48/45* haplotypes is shown for Mexico, Honduras, Brazil, Colombia, Myanmar, Thailand, South Korea, and North Korea. Haplotypes I–VII are shown in different *colours*

Comparison of *pvs48/45* polymorphic positions found in LA countries (Brazil, Colombia and Honduras) with the nine polymorphic positions reported for South Korea, North Korea, India and Indonesia showed a set of amino acid substitutions exclusively present among LA isolates. This set contained the substitution E353Q, which is the most frequent among LA isolates (41.9 %), together with the less frequent substitutions T108A (2.3 %), P264H (2.3 %), N324S (2.3 %), and K370N (2.3 %). On the contrary, D335Y, which is highly conserved among Asian isolates (95.5 %), was not detected among LA isolates. Amino acid variations H211N and K250N are found in both Asian and LA isolates. These two substitutions, highly conserved among Asian isolates (98.5 and 100 %, respectively) were only found in 18.6 and 39.5 % of *P. vivax* LA isolates, respectively. Other substitutions previously reported for Asian isolates [17, 18]: K26R (1.5 %), E35K (62.1 %), A367T (95.5 %), I380T (19.7 %), K418R (95.5 %) were not found in the present study. As a result, the number of haplotypes for worldwide samples was 18 (Table 3). There was variation of haplotype diversity geographically: low in North Korea, Peru and Mexico, and higher in Myanmar and Colombia. Nucleotide diversity (π) was 0.00173 for worldwide samples, ranging between 0.00058 and 0.00216 (Table 3). In Myanmar and Colombia π was relatively high, whereas it was low in Mexico, Peru and South Korea. A sliding window plot of π revealed a peak at nucleotide positions 1000–1160 in the CRD-3 region where four of the nine amino acid changes observed in Pvs48/45 occurred (Fig. 1b). The π of *Pv*48/45 in worldwide samples was at least one order of magnitude lower than that of known blood-stage antigen genes, such as *pvmsp1*, *pvmsp3a* and *pvdbp* (Fig. 2).

**Table 3:** Estimates of DNA sequence polymorphism and tests of neutrality for *pvs48/45*

Country	Total isolates	SV sites	PI sites	# Mutations	*K*	H	Hd ± SD	π ± SD	dN	dS	Reference
Mexico	15	0	2	2	0.441	2	0.221 ± 0.121	0.00033 ± 0.00033	0.00042	0.00000	This study
Honduras	12	3	2	5	1.128	4	0.445 ± 0.170	0.00123 ± 0.00094	0.00136	0.00077	This study
Colombia	60	3	4	7	1.929	9	0.841 ± 0.024	0.00188 ± 0.00073	0.00185	0.002	This study
Peru	24	1	2	3	0.372	3	0.218 ± 0.103	0.00034 ± 0.00046	0.00045	0.00000	This study
Brazil	13	2	1	3	0.59	4	0.526 ± 0.153	0.00058 ± 0.00062	0.00019	0.00194	This study
Latin America	124	3	5	8	1.228	11	0.610 ± 0.047	0.00168 ± 0.00087	0.00176	0.00151	This study
Myanmar	8	2	5	7	2.722	5	0.889 ± 0.071	0.00216 ± 0.00110	0.00282	0.0000	This study
Thailand	18	5	4	9	1.392	5	0.484 ± 0.138	0.00116 ± 0.00101	0.00103	0.00085	This study
South Korea	86	0	2	2	0.716	3	0.611 ± 0.026	0.00053 ± 0.00004	0.00069	0.00000	[17]
North Korea	46	2	7	9	1.987	5	0.0018 ± 0.042	0.00147 ± 0.00069	0.00029	0.00186	[18]
Worldwide	282	7	5	12	1.047	15	0.686 ± 0.00149	0.00173 ± 0.00149	0.00188	0.00149	This study

*SV* singleton variable, *PI* parsimony informative, *K* average number of pair-wise differences, H number of haplotypes, Hd haplotype diversity, π observed average pair-wise nucleotide diversity, dN number of non-synonymous substitutions, dS number of synonymous substitutions

### Geographic distribution of vs48/45 haplotypes among LA isolates

The wild-type (Sal I-like) haplotype predominated among Brazilian (92 %), Peruvian (90 %), Mexican (87 %), and Honduran (71 %) isolates, however in Colombian isolates, it was the second-most frequent haplotype (24 %). Colombian isolates showed a major presence of haplotype I (52 %), which was less frequent in Honduran isolates (14 %), and not detected among Brazilian, Peruvian and Mexican samples. Haplotype VI, the most frequent in Myanmar and Thailand, was found only among Colombian isolates, whereas haplotype VII was only present in Colombia isolates. Colombia showed the greatest haplotype diversity (0.841 ± 0.024) with eight non-Sal I haplotypes circulating in the country.

### Mapping of immune epitopes

A total of 12 linear epitopes were identified in regions 1, 5, 6, and 12, displaying greater algorithmic scores predictive of putative antigenic peptides (Table 4). A high score indicates maximum probability of immunogenicity in the host. Four of the 12 predicted regions contained polymorphic amino acids, however mutations were located in less antigenic epitopes. These substitutions did not affect the epitope prediction, and had limited impact on the calculated antigenic score (e.g., region 7, 1.154 vs 1.153).

**Table 4:** Potential antigenic determinants for the Pvs48/45

# Antigenic region	AA start	AA end	Length	Score	Polymorphism	Sequence^a^
1	4	19	16	1.235	None	RQLANLLLVLSLLRGI
2	46	64	19	1.083	None	GFKCNFSSKGVHNLEPILT
3	66	90	25	1.197	None	KRSLVCSIYSYFIYDKIKLTIPKKI
4	99	109	11	1.129	None	PEKCFQTVYTN
5	165	189	25	1.206	None	ISNVKGRVALVQVNVLKYPHKITSI
6	220	237	18	1.228	None	GELVVLACEKVDDKCFKK
7	242	304	63	1.154	K250N	SPLSLYKSKKIVYHKNLSIFKAPVYVKSADVTAECSCNVDSTIYTLSLKPVYTKKLIHGCNFS
8	326	341	16	1.138	D335Y	QITCSIELVDTSYNHL
9	347	361	15	1.167	E353Q	PGEVLPECFFQVYQR
10	366	377	12	1.141	A376T	LEPSKIVYLDAQ
11	394	404	11	1.152	None	IVKIFGLVGSI
12	422	447	26	1.255	None	YMSVKIAAGYFGFLAKIFILLIVLLL

^a^Italics AA indicates Cysteine residues, whereas Bold italics AA represent the location of polymorphic sites

### SNPs location on 3D Pvs48/45 domain structure

**Fig. 3:**
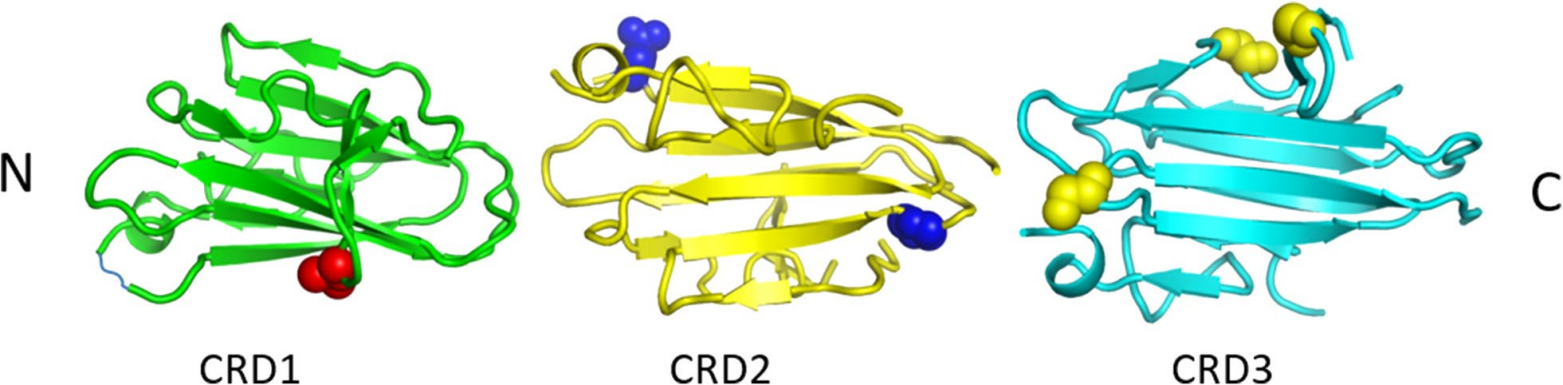
A ribbon representation of 3D models of the Pvs48/45 domains. Space-filling representation of amino-acid side chains (*red*, *blue* and *yellow balls*) denotes the locations of the non-synonymous amino acid substitutions in the three cysteine-rich domains (CRD)

A 3D model of Pvs48/45 was generated to understand the potential structure–function relationship of the nine polymorphic amino acids under balancing selection. SNPs were located in different regions of Pvs48/45: H211N (18.6 %) was in an intervening region between domains 1 and 2; K250N (39.5 %) was located in domain CRD-2, which is not considered a six-cys domain; and E353Q (41.9 %), A376T (50.7 %), K390T (1.1 %), and K418R (51.7 %) in domain CRD-3, which is the nearest to the trans-membrane region (Fig. 3). Mapping of the SNPs that led to amino acid substitutions onto the *Pvs48*/45 3D model domains showed that these residues are located in the loop regions.

## Discussion

This study showed a limited *pvs48/45* genetic diversity in a total of 282 *P. vivax* isolates worldwide. Only 18 amino acid substitutions were found within the entire protein sequence of an estimated 450 amino acid residues. *Plasmodium vivax* sequences corresponded to isolates from nine distant countries in three continents. Two *pvs48/45* haplotypes were found to be shared by Asian and LA parasites, with a strong geographical allele clustering. Recent studies on the diversity of *pvs48/45* among *P. vivax* isolates circulating in the Korean peninsula, China and Thailand showed low levels of genetic diversity [17, 18, 21, 37]; likewise, Brazilian isolates showed almost no variation when compared to the Sal I reference strain (PVX_083235), originally from El Salvador.

Interestingly, Korean, Colombian and Honduran isolates displayed limited sequence polymorphism. Pvs48/45 nucleotide and amino acid substitutions in isolates from Korea were found at pre-CRD (E35K), CRD-2 (H211N and K250N) and CRD-3 (D335Y, A376T, I380T, K418R). Pvs48/45 was more conserved in the Korean populations where nucleotide diversity varied from 0.00053 [17] to 0.00147 [17, 18], and the haplotypes V was unique for these populations. *Plasmodium vivax* was recently re-introduced into the peninsula of Korea with a rapid spread pattern, suggestive of a high genetic diversity [38]. This could explain the greater *pvs48/45* variability observed in Korean isolates as compared to isolates from Honduras and Mexico, and the almost absent variability among Brazilian isolates Despite the variability in the Korean isolates, globally, Pvs48/45 remains a highly conserved antigen compared to other transmission-blocking vaccine candidates.

Results are in agreement with previous observations that transmission-blocking vaccine candidate antigens Pvs25, Pvs28, Pvs48/45, and PvWARP showed limited sequence polymorphisms [18]. More importantly, the limited polymorphism found here does not appear to affect the immunogenicity of predicted epitopes as only three of nine amino acid substitutions in isolates from Colombia and Honduras were located at CRD2 (H211N and K250N) and CRD3 (E353Q). No substitutions were observed in CRD1, in agreement with reports for Korean isolates and for *P. falciparum* in Kenyan, Thai, Indian, and Venezuelan isolates [39, 40]. However, further studies are required to confirm the role of these B cell epitopes and to identify T cell epitopes in defining the potential of Pvs48/45 as a transmission-blocking vaccine candidate.

Pvs48/45 Cys domains are important for proper conformation of immune epitopes [8, 45]. In Pfs48/45, four epitopes designated epitope V (CRD1), epitope IIb (CRD2), epitope III (CRD2), and epitope I (CRD3) have been described previously [8]. The N-terminal CRD2 and CRD3 epitopes appear to be transmission-blocking targets as specific monoclonal antibodies to these regions have demonstrated an ability to prevent parasite fertilization, and consequently mosquito infection [12, 46].

Substitutions H211N and K250N are predicted to be located in loop regions [18], which could serve as potential vaccine targets. Interestingly, no amino acid substitutions were found in any of the Cys residues of Pvs48/45 in the present study, which are critical for proper presentation of the transmission-blocking epitopes [12]. However, an amino acid substitution (E353Q) was found next to a Cys residue reported to be involved in a disulfide bond. The influence of this variation on the formation of the disulfide bridge is yet to be explored.

Although parasite antigen diversity has been explained in part by immune pressure on the parasite [41–43], it does not appear to apply to Pvs48/45 as the protein is highly immunogenic, and it is expressed during the entire gametocyte maturation process. For *P. vivax* sexual antigens the exposure to the immune system could be longer than in *P. falciparum* due to the early appearance of *P. vivax* gametocytes in circulation [44]. However it does not appear to apply to Pvs48/45 as the protein is conserved and highly immunogenic under natural conditions.

Transmission-blocking vaccines have been considered a promising strategy/tool for malaria control/elimination. In *P. vivax* malaria it has been observed that gametocytogenesis occurs earlier than in *P. falciparum* and remains active even in asymptomatic carriers [44]. Consequently, malaria transmission to mosquitoes is likely to be more efficient, and thus, transmission-blocking vaccines would have a greater impact [47].

## Conclusions

There is limited sequence polymorphism in *pvs48/45* with noted geographical clustering among Asian and American isolates. The low genetic diversity of the protein does not influence the predicted antigenicity or protein structure and, therefore, supports its further development as a transmission-blocking vaccine candidate with widespread global potential.
